# Microbial diversity and antimicrobial resistance in faecal samples from acute medical patients assessed through metagenomic sequencing

**DOI:** 10.1371/journal.pone.0282584

**Published:** 2023-03-16

**Authors:** Maho Yokoyama, Leon Peto, Eric P. Budgell, Nicola Jones, Elizabeth Sheridan, Jane Liu, A. Sarah Walker, Nicole Stoesser, Hyun S. Gweon, Martin J. Llewelyn

**Affiliations:** 1 Department of Global Health and Infectious Diseases, Brighton and Sussex Medical School, Brighton, United Kingdom; 2 Nuffield Department of Medicine, University of Oxford, Oxford, United Kingdom; 3 Department of Infection, Oxford University Hospitals NHS Foundation Trust, Oxford, United Kingdom; 4 Department of Microbiology, University Hospitals Dorset, Bournemouth, United Kingdom; 5 Department of Microbiology, Royal United Hospitals Bath, Bath, United Kingdom; 6 School of Biological Sciences, University of Reading, Whiteknights, Reading, United Kingdom; 7 UK Centre for Ecology & Hydrology, Wallingford, Oxfordshire, United Kingdom; Bristol-Myers Squibb Company, UNITED STATES

## Abstract

Antimicrobial resistance (AMR) is a threat to global public health. However, unsatisfactory approaches to directly measuring the AMR burden carried by individuals has hampered efforts to assess interventions aimed at reducing selection for AMR. Metagenomics can provide accurate detection and quantification of AMR genes within an individual person’s faecal flora (their gut “resistome”). Using this approach, we aimed to test the hypothesis that differences in antimicrobial use across different hospitals in the United Kingdom will result in observable differences in the resistome of individual patients. Three National Health Service acute Hospital Trusts with markedly different antibiotic use and *Clostridioides difficile* infection rates collected faecal samples from anonymous patients which were discarded after *C*. *difficile* testing over a period of 9 to 15 months. Metagenomic DNA was extracted from these samples and sequenced using an Illumina NovaSeq 6000 platform. The resulting sequencing reads were analysed for taxonomic composition and for the presence of AMR genes. Among 683 faecal metagenomes we found huge variation between individuals in terms of taxonomic diversity (Shannon Index range 0.10–3.99) and carriage of AMR genes (Median 1.50 genes/cell/sample overall). We found no statistically significant differences in diversity (median Shannon index 2.16 (IQR 1.71–2.56), 2.15 (IQR 1.62–2.50) and 2.26 (IQR 1.55–2.51)) or carriage of AMR genes (median 1.37 genes/cell/sample (IQR 0.70–3.24), 1.70 (IQR 0.70–4.52) and 1.43 (IQR 0.55–3.71)) at the three trusts respectively. This was also the case across the sample collection period within the trusts. While we have not demonstrated differences over place or time using metagenomic sequencing of faecal discards, other sampling frameworks may be more suitable to determine whether organisational level differences in antibiotic use are associated with individual-level differences in burden of AMR carriage.

## Introduction

Antimicrobial resistance (AMR) is a global threat to public health [1] but obtaining representative data on the burden of AMR among different populations is challenging. Historically, data have been obtained using clinical sampling frameworks which are inevitably non-representative, usually do not assess AMR present in the commensal flora and involve culture-based detection which may not capture all relevant AMR mechanisms [2].

Metagenomic analyses, using short-read next-generation sequencing data, have the ability to quantify thousands of resistance genes in a single sample. Metagenomics can provide accurate detection and quantification of AMR genes within an individual person’s faecal flora (their gut “resistome”) [3]. This approach has been found to be superior to conventional methods for AMR surveillance [4] and could be used to provide a patient-level, near real-time assessment of different strategies intended to modify the risk of AMR selection, such as short vs long antibiotic treatment duration.

The term “antimicrobial stewardship” is used to describe organisational level strategies to optimise antimicrobial use and minimise selection for AMR, and studies which evaluate different stewardship strategies typically compare organisations rather than individual patients [5]. Even within national healthcare systems, such as the National Health Service (NHS) in England, antimicrobial use varies widely between hospitals, both in terms of Defined Daily Doses (DDDs) of antibiotics prescribed per patient and the relative use of broad-spectrum vs narrow-spectrum agents [6].

In this study, we aimed to test the hypothesis that substantial differences in hospital-level use of antibiotics will result in detectable differences in the faecal resistome of patients treated at those hospitals. If so, this would indicate that assessment of the faecal resistome could be an appropriate direct measure of harms associated with antibiotics in studies evaluating antimicrobial stewardship interventions. We therefore undertook metagenomic analyses of microbial diversity, AMR gene burden and AMR gene diversity in faecal samples submitted for *Clostridioides difficile* testing from unselected adult medical admissions to three large acute hospitals in England.

## Methods

### Setting

The study was performed as a sub-study of the Antibiotic Review Kit (ARK)–Hospital trial [7], registration ISRCTN12674243. Within the ARK trial, hospitals supplied routine electronic health data for acute/general medical inpatients, monthly antibiotic DDDs dispensed to acute medical admission areas (overall and by drug and route of administration), and *C*. *difficile* test results for the acute medical inpatients, as described previously [7]. Three ARK trial sites who were able to provide discarded stool samples after processing for *C*. *difficile* took part in the sub-study. Each was an acute NHS hospital trust based in different cities of southern England with distinct patterns of antimicrobial use, and sampling and data analysis was undertaken for distinct time periods for each Trust as defined in Table 1.

**Table 1:** Characteristics of participating sites.

	Trust A	Trust B	Trust C
Sampling time frame	July 2017-Aug 2018	Jan 2019-Mar 2020	Feb 2019-Oct 2019
Median monthly acute medical admissions (IQR)	5493 (5323–5643)	3572 (3451–3728)	2445 (2337–2541)
Median monthly total systemic antibiotic DDDs per acute medical admission (IQR)*	1.65 (1.48–1.69)	3.31 (3.08–3.66)	0.48 (0.46–0.51)
Median monthly percent of antibiotics used in Access category (IQR)	35.7% ( 34.7–36.7)%	64.3% (62.9–65.2)%	62.2% (57.7–68.8)%
*Median monthly C*. *difficile* colonisations (inclusive of infection)/100 acute medical admissions**	0.50 (0.44–0.55)	0.50 (0.44–0.54)	0.34 (0.24–0.39)

* DDDs are a World Health Organisation system for standardising antibiotic usage. 1 DDD reflects the average daily maintenance dose of an antibiotic when used in adults for its main indication. **. *C*. *difficile* colonisation defined as positive testing by PCR or Glutamate Dehydrogenase. Infection rates not shown because toxin test results defining of infection were not available for one site.

Overall use of systemic antibiotics was defined by DDDs per acute medical admission and antibiotics were categorised using the World Health Organisation AWaRe categorisation adapted for use in England [8]. The percentage of “Access” antibiotics (those which should be used as first-line to preserve antibiotics in the Watch and Reserve categories) were summarised for each site. For each site the number of acute medical admissions with a stool sample testing positive for presence of *C*. *difficile* (within -1/+90 days of admission) was calculated per 100 acute medical hospital admissions per month.

### Sampling framework

Microbiology laboratories at the three participating hospitals sent a randomly selected sample of stool samples from adult acute medical inpatients left over after routine testing for *C*. *difficile* infection. The only meta-data available for samples was therefore the date of sampling and hospital site. This approach was taken to avoid the burden of, and biases inherent in, taking informed consent. For each site samples were provided determined by the time of their involvement in the ARK-hospital cluster randomised controlled and availability of clinical laboratory staff to process samples and this varied between Jul 2017 and Oct 2019 (S1 Table). The only metadata available were date of sampling and site. Anonymised testing within the sub-study was approved as part of the main trial protocol by the South Central Oxford C Research Ethics Committee (REC) (17/SC/ 0034) and the Confidentiality Advisory Group (17/CAG/0015) without individual patient consent.

### DNA extraction and sequencing

**Fig 1:**
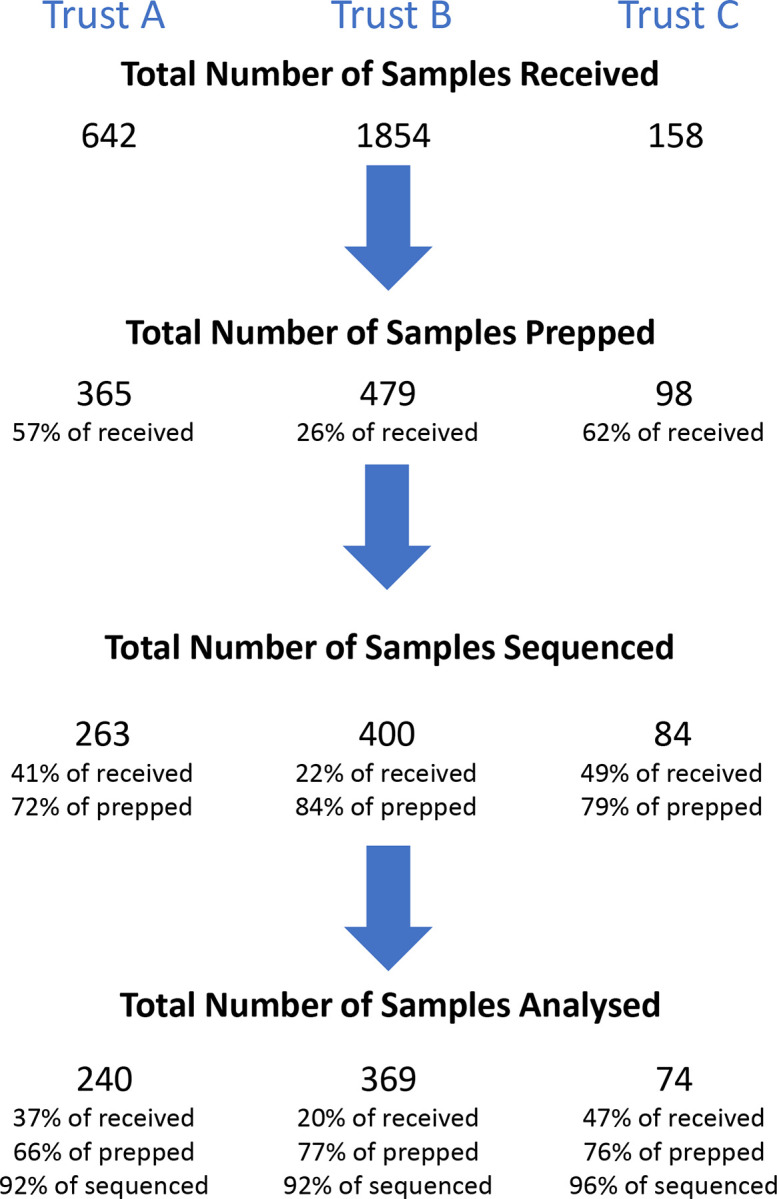
Sample processing flow-diagram.

Samples were shipped on dry ice and stored at -80°C prior to DNA extraction. The number of samples received from each site per calendar month varied widely (S1 Table). For logistic and cost reasons, not all samples received from some timepoints could be processed through to sequencing therefore where more samples were available than could be analysed samples were selected for processing at random aiming to ensure where possible a minimum of 10, ideally 15 sequenced samples were available for analysis in each month (Fig 1, S1 Table).

To obtain metagenomic DNA from the samples, DNA extraction was carried out using the QIAamp Fast DNA Stool Mini kit (Qiagen, Hilden, Germany) according to the manufacturer’s protocol with additional steps and modifications as follows: A known concentration of *Thermus thermophilus* DNA, strain DSM579 (DSMZ, Braunschweig, Germany), either 103 ng or 20 ng, was added to Stool Transport And Recovery (S.T.A.R.) Buffer (Roche, Basel, Switzerland). 1 ml of the *T*. *thermophilus*-S.T.A.R buffer was pipetted into Lysing Matrix E tubes (MP Biomedicals, Santa Ana, USA) and weighed to the nearest mg to obtain the tube weight. Faecal sample was added to the weighed Lysing Matrix E tubes with the *T*. *thermophilus*-S.T.A.R. buffer and then weighed again to the nearest mg to obtain the sample weight. The samples were then bead beaten using Tissue Lyser LT (Qiagen) at 50 Hz for 10 mins. 1 ml of the bead beaten sample was then transferred to the InhibitEX buffer from the QIAamp Fast DNA Stool kit and heated at 95°C for 5 mins, and then centrifuged at 13,000 rpm for 3 mins. 600 μl of the supernatant from the above InhibitEX step was added to 45 μl proteinase K, then 600 μl Buffer AL was added to the mix. The kit protocol was then followed; briefly, the above mix was incubated at 70°C for 10 mins, before 600 μl ethanol was added. The resulting lysate was loaded onto a spin column, then the columns were washed with 500 μl each Buffer AW1 and Buffer AW2. The bound DNA was eluted using 100 μl molecular grade water before the DNA concentration was determined using Qubit dsDNA HS Assay (Invitrogen, Waltham, USA). A number of samples could not be taken to the next step due to insufficient DNA concentration (Fig 1).

Samples were sequenced to a minimum depth of 10 million reads (including human and non-human). Sequencing was conducted by the Oxford Genomics Centre (Oxford, UK) using their High Multiplex Whole Genome/MMM library preparation for sequencing on a NovaSeq 6000 platform (Illumina), generating 150 bp paired-end reads.

### Data processing and analysis

Taxonomic classification was performed using Kraken2 [9], and samples with more than 7.5 million sequences classified as non-human were selected for downstream analysis (Fig 1, S1 Table). To address the difference in sequencing effort across samples which varied between 10 to 40 million sequences, all samples were randomly subsampled to 10 million sequences [10,11]. Samples were subsequently processed using a published methods implemented within ResPipe (v.1.5.0) [12]. Briefly, AMR gene counts were generated for sequences that mapped with 100% sequence identity against Comprehensive Antibiotic Resistance Database v.3.0.9 [13]. The resulting table of AMR gene profiles for all samples was normalised using a technique called Fragments Per Kilobase Million (FPKM); briefly, the relative abundance of each AMR gene was multiplied by 1,000,000 to give a “Fragment/Reads Per Million” (FPM). Next, to account for the variation in AMR gene lengths, the FPMs of each AMR gene were divided by its length in kb resulting in FPKM. Finally, the FPKM values were divided by the average count of 31 single copy genes found in each sample to yield “AMR genes per bacterial cell”. For the purpose of defining presence or absence of AMR genes, a sample was considered to contain an AMR gene if the FPKM value was >0.1. Individual AMR genes were then grouped by inferred resistance to antibiotic classes or class gene families. Quantification of diversity metrics was performed using the vegan R package (v.2.5–7 in R v.4.1.2) [14].

### Statistical analysis

Differences in species diversity and AMR gene carriage were tested for statistical significance by pair-wise Chi-squared tests and reported when P<0.05.

## Results

A total of 683 metagenomes were included in the analysis from 2,654 samples in total, a median of 18 per site per calendar month (interquartile range (IQR)15-22, range 0–42) (S1 Table). For site A samples were available July 2017-August 2018, for site B January 2019-March 2020 and site C February 2019-October 2019. The median number of reads from each sample which was sequenced was 15,981,463 (IQR 13,995,644–18,584–776). Of the 683 samples analysed, the number of reads which mapped to AMR genes ranged from 272 to 179,190 per sample (IQR 4,022–24,068, median 9,510).

### Species diversity

**Fig 2:**
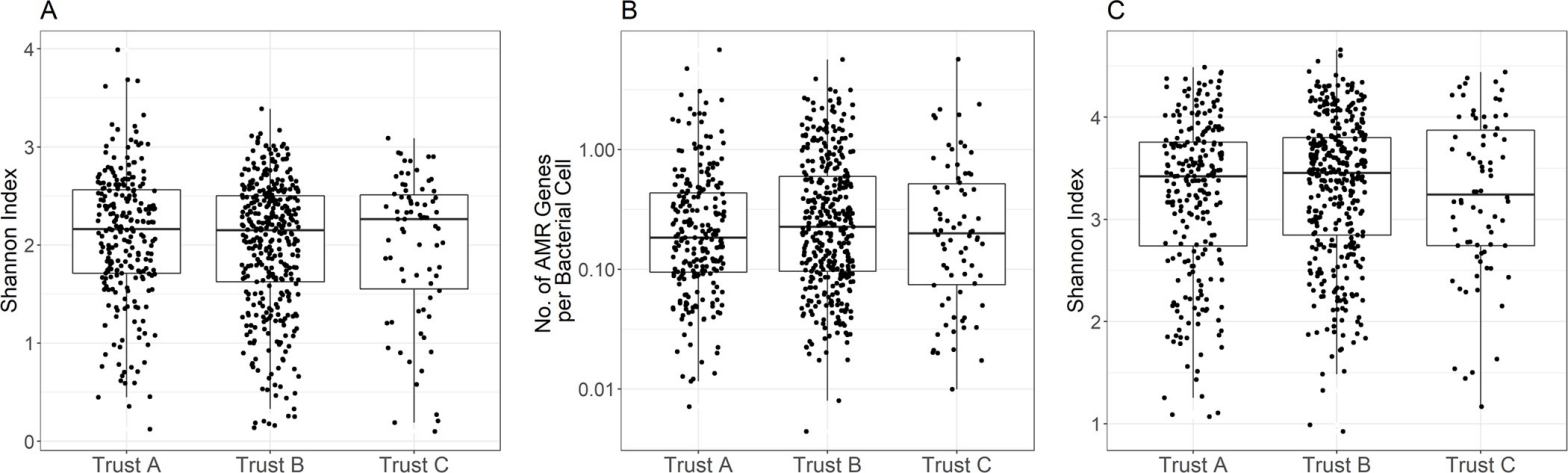
Species diversity and AMR resistance burden among faecal samples by hospital site. A) species alpha diversity, B) AMR gene carriage, C) resistome alpha diversity.

The overall species alpha-diversity of samples varied widely (Shannon Index range 0.10–3.99) but the median and IQR were very similar at the three hospitals: median 2.16 (IQR 1.71–2.56), 2.15 (IQR 1.62–2.50) and 2.26 (IQR 1.55–2.51) for sites A, B and C respectively (Fig 2A). There was no consistent variation in median or IQR of diversity over time (S1 Fig).

### AMR burden and diversity

The overall burden of AMR genes per bacterial cell in faecal samples also varied widely from 0.03–48.55 with a median of 1.50 genes per cell per sample overall. The median (IQR) AMR genes carried were very similar at the three hospitals: median 1.37 (IQR 0.70–3.24), 1.70 (IQR 0.70–4.52) and 1.43 (IQR 0.55–3.71) for sites A, B and C respectively (Fig 2B). There was no consistent variation in median or IQR AMR gene carriage over time (S2 Fig).

The alpha-diversity present within the faecal resistome was broadly comparable to the overall species alpha-diversity, ranging from 0.62–4.46. Again, there was no evidence of variation between sites, with median 3.17 (IQR 2.63–3.54), 3.17 (IQR 2.74–3.56) and 3.05 (IQR 2.64–3.58) for sites A, B and C respectively (Fig 2C). There was also no evidence of a relationship between diversity and calendar month of sampling (S3 Fig).

**Fig 3:**
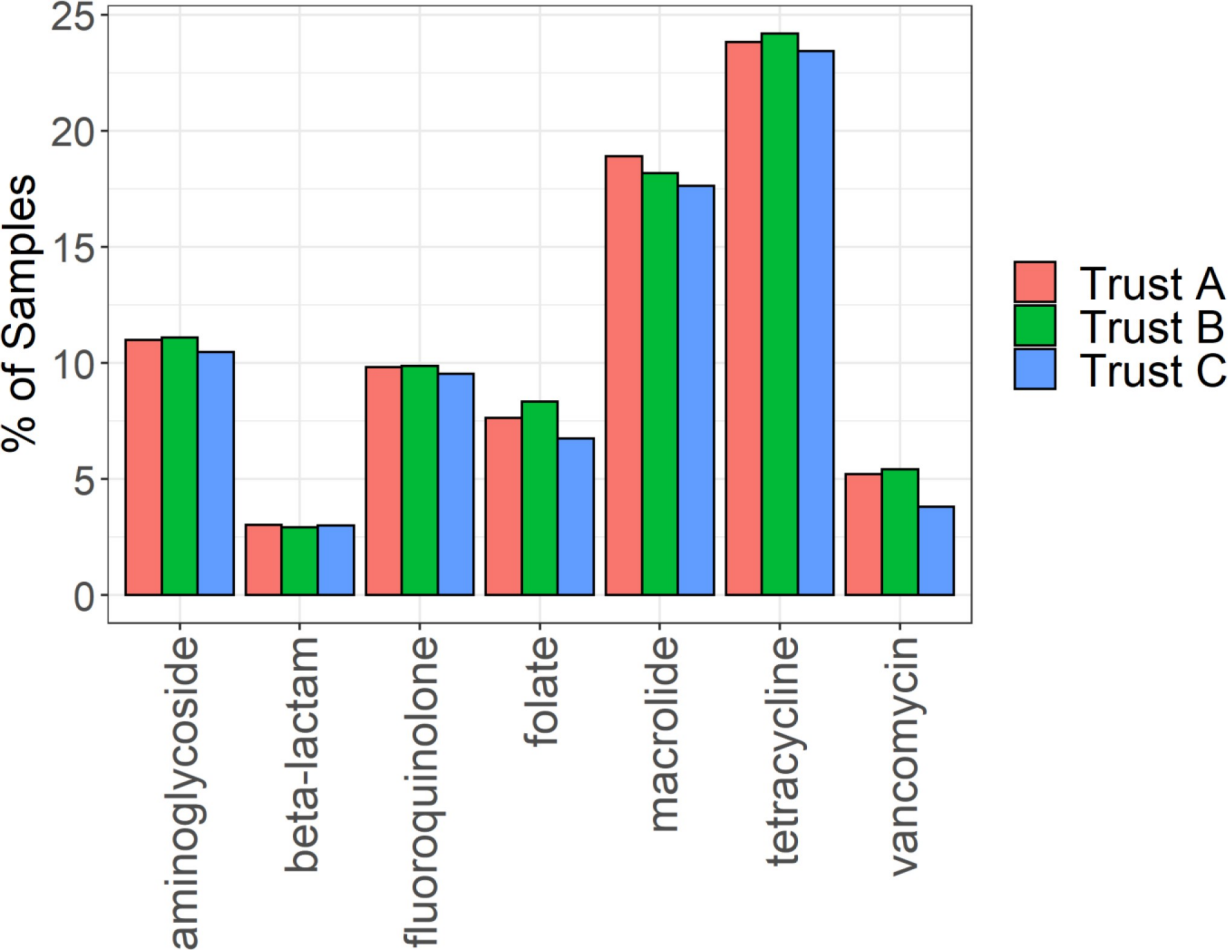
Frequency of resistance to major antibiotic classes identified in faecal samples. Graphs show the percentage of samples from each hospital site in which resistance to each antibiotic class was identified by metagenomic sequencing.

**Fig 4:**
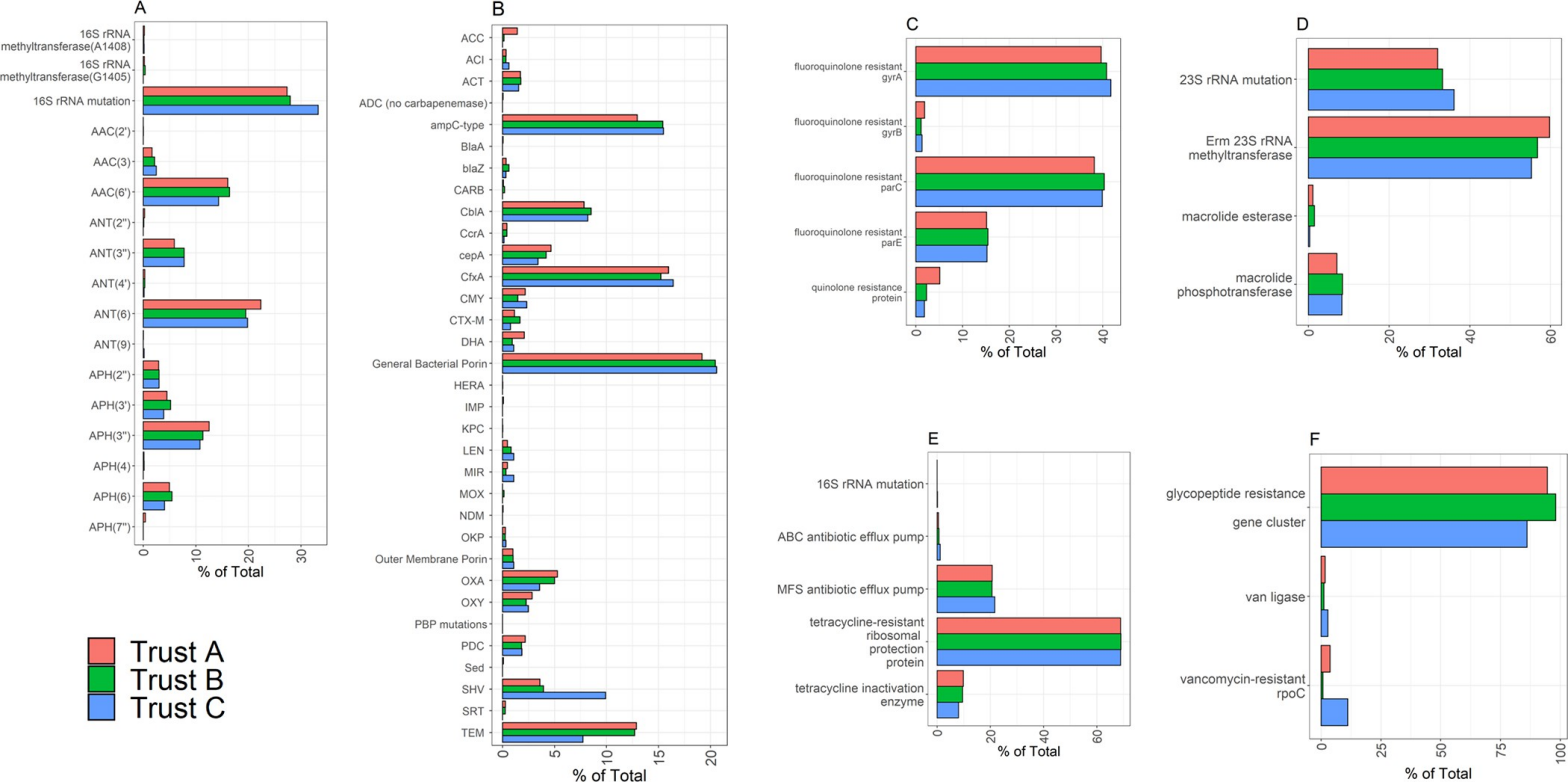
AMR gene frequency. Graphs show the contribution of different molecular mechanisms of resistance for each antibiotic class. A) aminoglycosides B) beta-lactams C) quinolones, D) macrolides, E) tetracyclines, F) glycopeptides.

In terms of resistance to major antibiotic classes, there was no evidence that the percentage of samples with genes conferring resistance to each antibiotic class varied across the three sites (Fig 3). This remained the case when different FPKM values were used as a cut-off to denote presence or absence of AMR genes (S4 Fig). Hence, a cut-off of 0.1 FPKM was chosen to balance positives and potentially false positive detections. We also considered the contributions of individual genotypes conferring resistance (Fig 4); although some numerical imbalances were apparent for a small number of comparisons (e.g. for SHV and TEM beta-lactamases) again there was no statistical evidence for association.

## Discussion

Here, we have conducted metagenomic analysis of 683 faecal metagenomes sequenced to median depth of ~16 million reads obtained from specimens submitted for *C*. *difficile* testing in adult medical patients receiving treatment at three large acute hospitals in England. This has allowed us to characterise the diversity and burden of AMR gene carriage among these patients but also make comparisons across time and place in a way that has not been attempted in humans previously. The most striking findings from our analyses are firstly the huge variation that exists in AMR gene diversity between patient samples and secondly the overall consistency of inpatient resistomes over time and place in terms of the burden and nature of AMR gene carriage.

Our estimates of overall and AMR resistance gene alpha diversity are consistent with previous studies of healthy individuals [15,16]. However, despite such marked inter-individual variation, other studies focusing on patients with chronic disease [17,18], different health states [19] and antibiotic exposure [20] have demonstrated detectable impact of these conditions and antibiotic prescribing on the faecal metagenome and resistome. At the three hospitals from which we took samples, hospital-level antibiotic dispensing to general medical admission areas varied markedly in terms of both intensity of use (DDDs per acute medical admission) and nature of use (proportion of “Access” antibiotics). For example, Trust C had low antibiotic use overall, a predominance of Access agents, and, in keeping with previous studies, a low rate of *C*. *difficile* infection. Nevertheless, we found no suggestion in our data of any detectable hospital-level difference in diversity or AMR gene carriage in the faecal samples obtained from patients.

Our study has important limitations. We generated metagenomic data on a very limited number of admitted patients (<1%/month), and it is possible that pooling larger numbers of samples to evaluate the wider inpatient-level resistome might better reflect hospital-level prescribing, that the impact of prescribing on faecal metagenomes can be more reliably ascertained in certain population sub-groups or that there is a threshold effect through which above a certain level of antibiotic use differences are small. Our samples were uncoupled from any associated metadata including age, gender, ethnicity, medical history, previous antibiotic exposure and time from admission, all of which may influence AMR gene burden. If samples were taken from patients recently admitted to hospital then the faecal resistome may potentially be more likely to reflect community antibiotic use, for which data are not available, rather than hospital prescribing. Similarly, some samples may have been taken from patients who were not on antibiotics. Notably however, the samples analysed were discards after *C*. *difficile* testing and therefore likely to be biased towards patients with recent antibiotic exposure, and towards a sub-group of patients with diarrhoea which might have also impacted on taxonomic and resistance gene distributions. While this has the potential to dilute our ability to measure the individual patient-level impact of antibiotic exposure, it would not be expected to detract from the validity of our comparisons which are of organisation-level antibiotic use and *C*. *difficile* colonisation rates. Site-related differences in resistance carriage might plausibly be smaller in such patients than in the wider population. Finally, the sensitivity of metagenomic data for the profiling of AMR genes in polymicrobial samples has been shown to be closely linked to sequencing depth [12], and we may therefore not have detected AMR genes present at very low frequencies but nevertheless clinically relevant, and ready to emerge under antibiotic selection. Strengths of our study include a multi-centre approach and monthly sampling, to ensure capture of any temporal signal.

In summary, we found no evidence that evaluating the faecal resistome of <1% of hospital inpatients using discarded samples submitted for *C*. *difficile* testing could be used to assess the impact of antibiotic practice on carried resistance at an hospital-level. Further studies investigating whether more detailed sampling, or pooled sampling, would be of benefit in detecting prescribing signatures might be considered.

## Supporting information

S1 Fig Species diversity in faecal samples from three hospital sites over time. (PDF)

S2 Fig AMR gene frequency in faecal samples from three hospital sites over time. (PDF)

S3 Fig Resistance gene diversity in faecal samples from three hospital sites over time. (PDF)

S4 Fig Frequency of resistance to major antibiotic classes identified in faecal samples and impact of changes in cut-off value. (PDF)

S1 Table Samples received and sequenced in the study. (PDF)
