# Supplementary figures and images for: Microbial diversity and antimicrobial resistance in faecal samples from acute medical patients assessed through metagenomic sequencing
Source: PLoS One. 2023 Mar 16;18(3):e0282584. doi: 10.1371/journal.pone.0282584 (PMC10019653; doi:10.1371/journal.pone.0282584)

S1 Figure. Species diversity in faecal samples from three hospital sites over time.

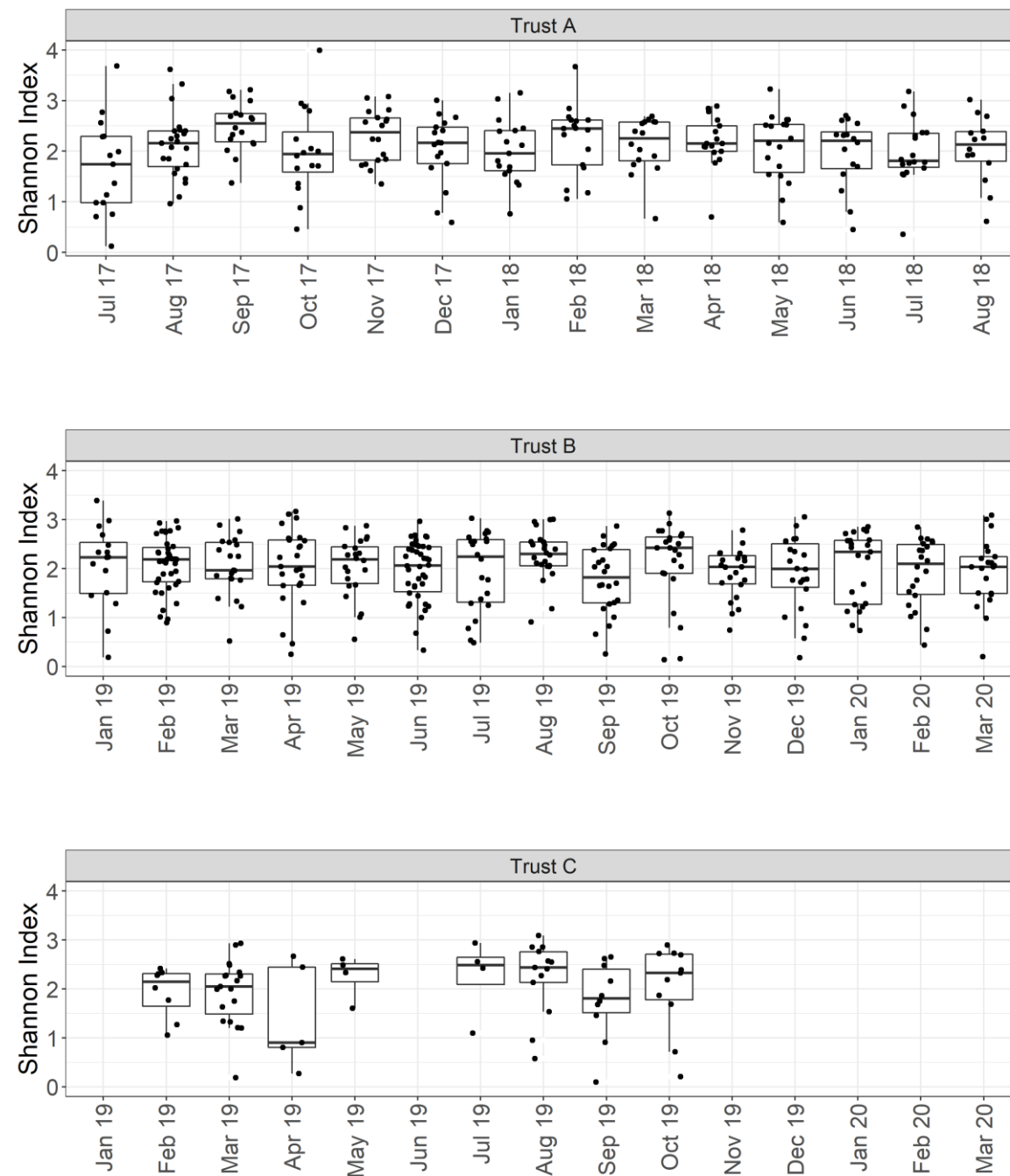

Supplement: S1 Fig — (PDF) [file pone.0282584.s001.pdf]

S2 Figure 2. AMR gene frequency in faecal samples from three hospital sites over time.

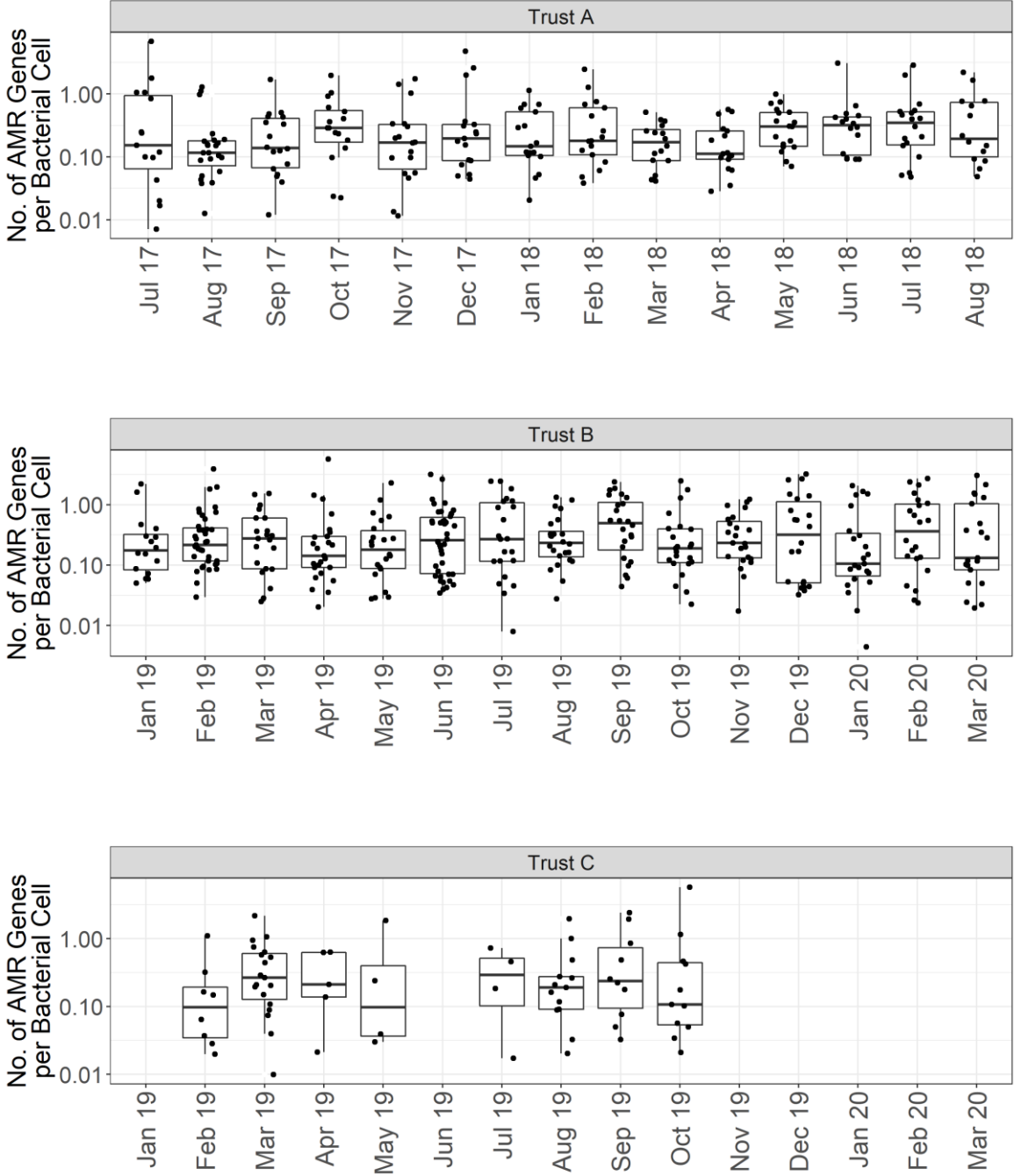

Supplement: S2 Fig — (PDF) [file pone.0282584.s002.pdf]

S3 Figure. Resistance gene diversity in faecal samples from three hospital sites over time.

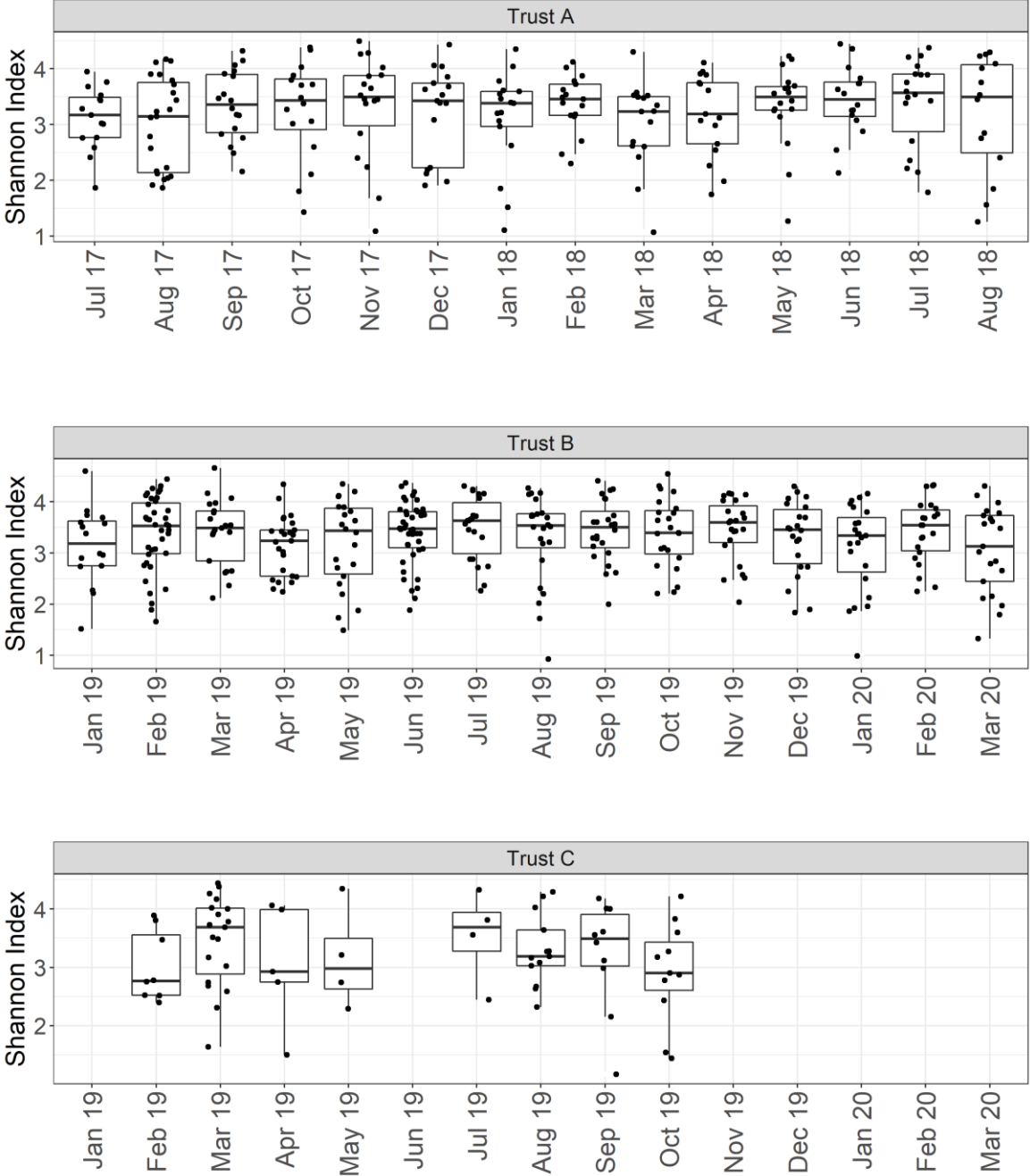

Supplement: S3 Fig — (PDF) [file pone.0282584.s003.pdf]
