# Supplementary material for: Microbial diversity and antimicrobial resistance in faecal samples from acute medical patients assessed through metagenomic sequencing
Source: PLoS One. 2023 Mar 16;18(3):e0282584. doi: 10.1371/journal.pone.0282584 (PMC10019653; doi:10.1371/journal.pone.0282584)
Supplement: S4 Fig — (PDF) [file pone.0282584.s004.pdf]

S4 Figure.
Frequency of resistance to major antibiotic classes identified in faecal samples and impact of changes in cut-off value.

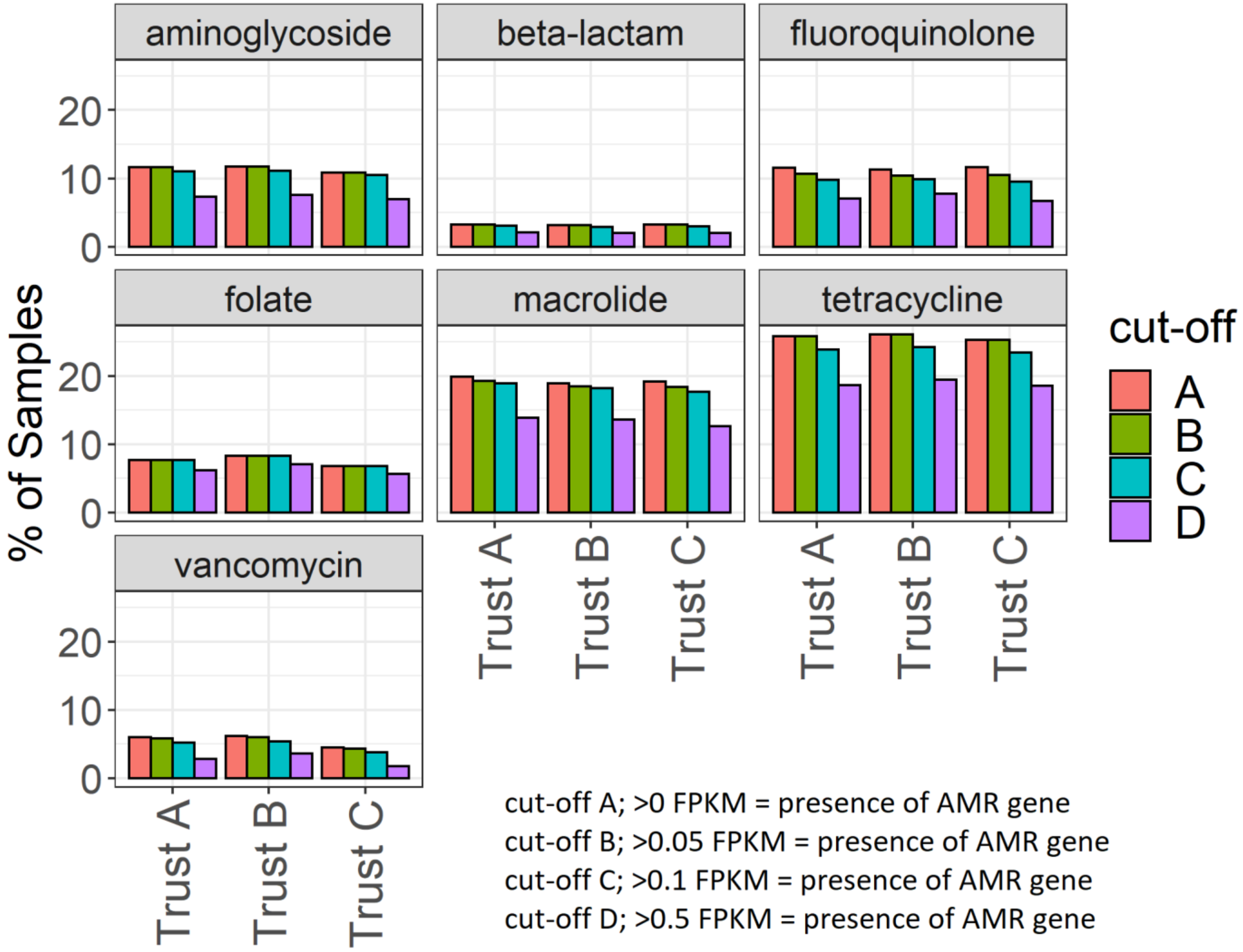

cut-off A; >0 FPKM = presence of AMR gene
cut-off B; >0.05 FPKM = presence of AMR gene
cut-off C; >0.1 FPKM = presence of AMR gene
cut-off D; >0.5 FPKM = presence of AMR gene
