# Supplementary material for: Microbial diversity and antimicrobial resistance in faecal samples from acute medical patients assessed through metagenomic sequencing
Source: PLoS One. 2023 Mar 16;18(3):e0282584. doi: 10.1371/journal.pone.0282584 (PMC10019653; doi:10.1371/journal.pone.0282584)
Supplement: S1 Table — (PDF) [file pone.0282584.s005.pdf]

S1 Table. Samples received and sequenced in the study

month	Trust A				Trust B				Trust C			
	total received	number prepped	number sequenced	number used for analysis	total received	number prepped	number sequenced	number used for analysis	total received	number prepped	number sequenced	number used for analysis
Jul-17	25	25	16	15								
Aug-17	41	36	23	23								
Sep-17	37	26	18	18								
Oct-17	37	23	18	16								
Nov-17	68	25	18	18								
Dec-17	46	23	18	17								
Jan-18	43	26	18	17								
Feb-18	49	23	18	17								
Mar-18	69	22	18	16								
Apr-18	54	23	18	17								
May-18	45	24	19	18								
Jun-18	48	33	20	16								
Jul-18	35	31	21	18								
Aug-18	45	25	20	14								
Jan-19					66	25	19	16				
Feb-19					83	58	42	39	10	10	9	8
Mar-19					142	25	23	21	88	28	21	19
Apr-19					200	27	26	25	7	7	5	5
May-19					204	24	23	22	7	7	6	4
Jun-19					130	55	43	42	0	0	0	0
Jul-19					205	48	24	22	5	5	4	4
Aug-19					225	26	24	24	18	18	17	13
Sep-19					150	29	25	24	11	11	11	10
Oct-19					81	31	26	23	12	12	11	11
Nov-19					81	24	24	23				
Dec-19					81	24	24	22				
Jan-20					81	30	27	23				
Feb-20					81	25	25	22				
Mar-20					44	28	25	21				
total	642	365	263	**240**	1854	479	400	**369**	158	98	84	**74**
